# La grossesse gémellaire sur un utérus pseudo unicorne: à propos d'un cas

**DOI:** 10.11604/pamj.2015.22.330.8011

**Published:** 2015-12-03

**Authors:** Hafsa aheri, Hanane Saadi, Saad Benkirane, Ahmed Mimouni

**Affiliations:** 1 Service de Gynécologie-Obstétrique, CHU Mohammed VI, Oujda, Maroc

**Keywords:** Grossesse gémellaire, malformation utérine, utérus unicorne, corne rudimentaire, rupture utérine, twin pregnancy, uterine malformation, horned uterus, rudimentary horn, uterine rupture

## Abstract

L'incidence des malformations utérines est estimée dans la population générale entre 0,1 et 3%. La survenue d'une grossesse en cas de malformation utérine est une situation potentiellement à haut risque obstétrical. Nous décrivons un cas très rare d'une grossesse gémellaire sur un utérus pseudo unicorne découverte précocement à 8 SA, permettant la réalisation d'une hémihysterectomie de la corne rudimentaire prévenant ainsi le risque majeur de la rupture utérine. L’évolution était favorable jusqu’à 37 SA où la patiente a été programmée pour césarienne prophylactique. Le dépistage échographique au premier trimestre de la grossesse est donc primordial permettant la détection systématique de ce genre de malformation afin de prévenir les complications.

## Introduction

La grossesse sur corne utérine rudimentaire d'un utérus pseudo-unicorne est un événement rare, de diagnostic difficile, pouvant être révélé sur un mode aigu secondairement à une rupture utérine, mettant alors en jeu le pronostic vital fœtal et maternel. Nous rapportons le cas d'une grossesse gémellaire sur un utérus pseudo unicorne découverte à 8 SA avec une évolution favorable après hémihysterectomie de l'hémimatrice borgne.

## Patient et observation

**Figure 1:**
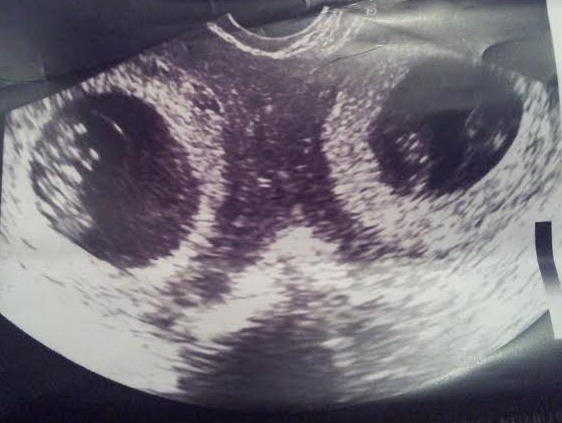
Echographie obstétricale endovaginale montrant un utérus malformé avec 2 hémimatrices non communicantes, chacune siège d'un sac gestationnel avec embryon de 8 SA avec activité cardiaque positive

**Figure 2:**
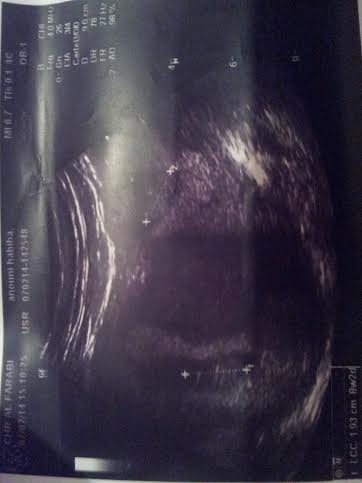
Echographie obstétricale sus-pubienne montrant un utérus malformé avec 2 hémimatrices non communicantes, chacune siège d'un sac gestationnel avec embryon de 8 SA avec activité cardiaque positive

**Figure 3:**
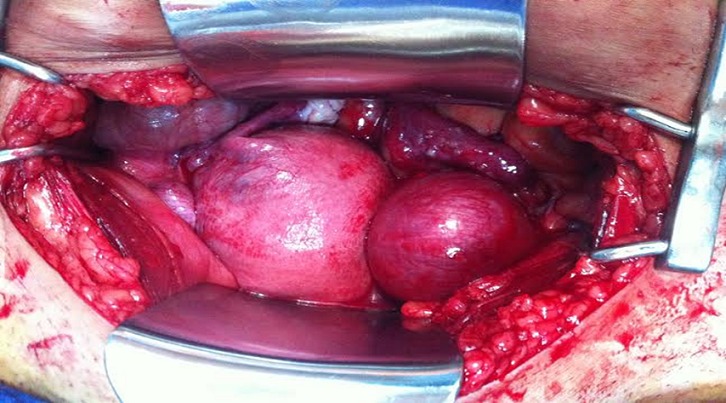
Utérus malformé avec 2 cavités gravides

**Figure 4:**
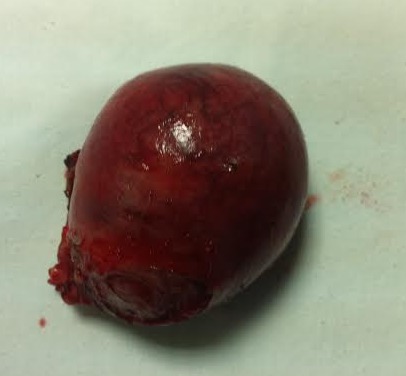
Pièce de l'hemihysterectomie (la corne rudimentaire)

**Figure 5:**
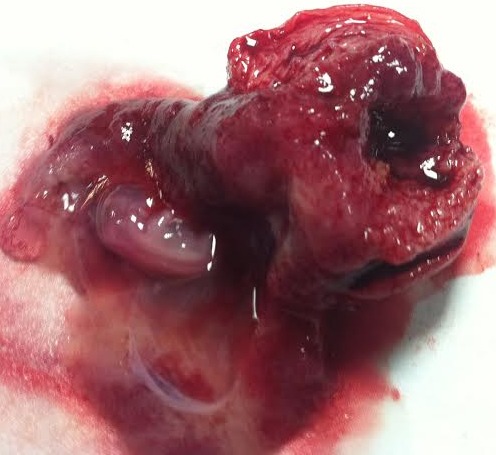
Ouverture de la corne rudimentaire: sac gestationnel avec embryon de 8 SA

**Figure 6:**
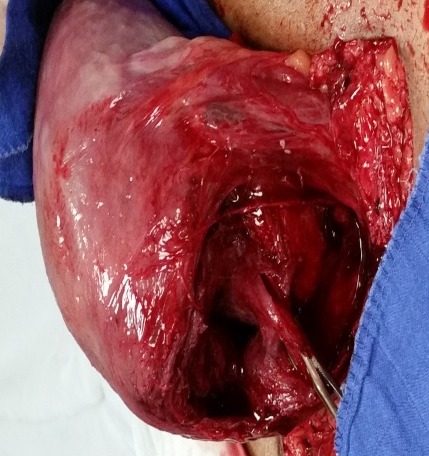
Hystérotomie basse segmentaire lors de la césarienne programmée. on note un bon état local

Il s'agit d'une patiente de 33 ans, grande multipare, ayant 4 enfants vivants accouchés par voie basse, se présente au service des urgences gynécologiques dans un tableau de douleurs pelviennes diffuses avec des métrorragies de faible abondance dans un contexte d'aménorrhée de 2 mois. L'examen trouve un abdomen souple avec une sensibilité de la fosse iliaque gauche. Au speculum: un col gravide unique. Au TV un utérus augmenté de taille, sans masse latero utérine. Une échographie obstétricale réalisée a objectivé un utérus malformé avec 2 hémimatrices non communicantes, chacune siège d'un sac gestationnel avec embryon de 8 SA avec activité cardiaque positive(Figure 1, Figure 2). Une c'lioscopie diagnostic a été réalisée objectivant le diagnostic d'un utérus pseudo unicorne. Une hémihysteréctomie de l'hémimatrice borgne a été réalisée par laparotomie sans franchir la deuxième cavité. (Figure 3, Figure 4, Figure 5). La patiente a été mise sous observation clinique et échographique avec une bonne évolution. A 37SA, on a réalisé une césarienne programmée permettant l'extraction d'un nouveau-né de sexe masculin avec un poids de naissance 3300g. L'exploration a montré un bon état local et une bonne cicatrisation de la corne utérine (Figure 6).

## Discussion

**Figure 7:**
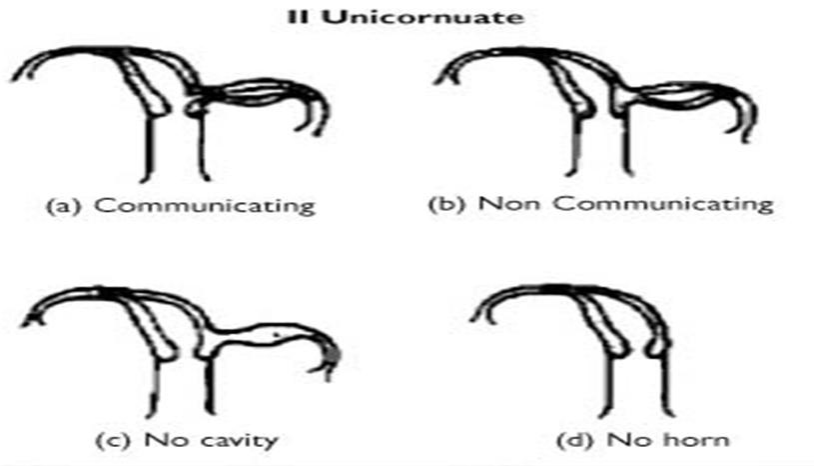
Classification AFS des utérus unicornes

Les utérus unicornes représentent 10% des malformations utérines [1]. Leur incidence, bien que difficile à préciser, est estimée à un pour 1000 femmes. La survenue d'une grossesse dans une corne utérine rudimentaire est une situation rare dont l'incidence est évaluée de 1/100 000 à 1/140 000 [2], et elle résulte de la migration intrapéritonéale des spermatozoïdes ou de l'ovocyte fécondé. Dans 10% des cas la corne rudimentaire communique avec l'utérus unicorne [3] alors que les cornes rudimentaires non communicantes et avec cavité représentent 36% [1]. Cette cavité est parfois tapissée d'un endomètre fonctionnel, exposant au risque d'anomalies de la placentation [1, 4]. Heinonen observa, sur une série de sept grossesses implantées dans la corne utérine rudimentaire, la présence de trois placentas accretas (43%) [1]. Dans notre observation, la patiente présente un utérus pseudo unicorne avec corne rudimentaire non communicante. Cette malformation utérine correspond au stade IIb de la classification de l'AFS (American Fertility Society) Figure 7. Les utérus pseudo-unicornes résultent d'un arrêt de développement de l'un des deux canaux de Müller avant qu'il n'atteigne le sinus urogénital entre la sixième et la neuvième semaine de développement embryonnaire: le côté aplasique donne donc naissance à une corne utérine rudimentaire. Il est rapporté que la corne rudimentaire est préférentiellement située à droite (62%) du faite que le canal de Müller gauche progresse plus caudalement que le droit [5]. Dans notre cas la corne était à gauche comme dans les cas rapportés par Daskalakis [6] et Kuscu [7].

Les anomalies de l'arbre urinaire sont fréquemment associées à cette malformation utérine (38%) et sont dominées par les agénésies rénales unilatérales toujours homolatérales au côté de la corne borgne [1]. Ce n’était pas le cas dans notre observation. Dans la littérature, 9 cas de grossesses gémellaires dans un utérus unicorne ont été publiés depuis 1945. Parmi ces cas, on retrouve 3 cas de grossesse gémellaire avec 1 jumeau développé dans chacune des 2 hemimatrices. Dans un cas, la grossesse s'est spontanément arrêtée dans la corne rudimentaire à 23 SA alors qu'elle se poursuivait jusqu’à 38SA dans l'utérus unicorne [8]. Dans le deuxième cas, la corne rudimentaire s'est rompue à 19 SA entraînant la perte des 2 fœtus [9]. Dans le troisième cas, le premier fœtus a été extrait par césarienne à 28 SA et le deuxième 8 j après, consécutivement à la rupture de la paroi utérine et de la corne [10]. EJNES a décrit pour la première fois le cas d'une grossesse gémellaire avec un embryon dans un utérus unicorne et un embryon dans une corne rudimentaire maintenue jusqu’à 29 SA, avec naissance en dehors de toute complication de 2 enfants vivants [11]. La complication majeure de ces grossesse est représentée par la rupture de la corne rudimentaire 90%, le plus souvent au deuxième trimestre de la grossesse, entrainant un tableau d'inondation péritonéale voire un état de choc maternel; cette situation est grevée d'une mortalité maternelle estimée à 0.5% et un taux de sauvetage fœtal de l'ordre de 2% [5]. Le traitement repose sur l'extraction fœtale et la résection de la corne rudimentaire et de la trompe homolatérale, afin de prévenir le risque de grossesse tubaire.

## Conclusion

La présence d'un utérus pseudo-unicorne avec corne rudimentaire non communicante est une source importante de complications gynéco-obstétricales. Le pronostic des grossesses associées à cette malformation est le plus souvent défavorable, d'où l'importance de l’échographie obstétricale du premier trimestre qui permet de faire le diagnostic de la grossesse intra utérine mais aussi de vérifier l'absence de malformation utérine.
